# Soluble Proteoglycans and Proteoglycan Fragments as Biomarkers of Pathological Extracellular Matrix Remodeling

**DOI:** 10.1002/pgr2.70011

**Published:** 2024-11-16

**Authors:** Marsioleda Kemberi, Alexander F. Minns, Salvatore Santamaria

**Affiliations:** ^1^ Barts and the London School of Medicine and Dentistry Queen Mary University of London London England UK; ^2^ Department of Biochemical Sciences School of Biosciences, Faculty of Health and Medical Sciences, University of Surrey Guildford Surrey UK

**Keywords:** ADAMTS, aggrecan, matrix metalloproteinases, neoepitope antibodies, proteases, proteoglycans, shedding, syndecans, versican

## Abstract

Proteoglycans and their proteolytic fragments diffuse into biological fluids such as plasma, serum, urine, or synovial fluid, where they can be detected by antibodies or mass‐spectrometry. Neopeptides generated by the proteolysis of proteoglycans are recognized by specific neoepitope antibodies and can act as a proxy for the activity of certain proteases. Proteoglycan and proteoglycan fragments can be potentially used as prognostic, diagnostic, or theragnostic biomarkers for several diseases characterized by dysregulated extracellular matrix remodeling such as osteoarthritis, rheumatoid arthritis, atherosclerosis, thoracic aortic aneurysms, central nervous system disorders, viral infections, and cancer. Here, we review the main mechanisms accounting for the presence of soluble proteoglycans and their fragments in biological fluids, their potential application as diagnostic, prognostic, or theragnostic biomarkers, and highlight challenges and opportunities ahead of their clinical translation.

**Abbreviations**
AD: Alzheimer's disease
ADAM: a disintegrin‐like and metalloproteinase
ADAMTS: a disintegrin‐like and metalloproteinase with thrombospondin motifs
AIS: acute ischemic stroke
CAD: coronary artery disease
CCC: coronary collateral circulation
CKD: chronic kidney disease
COPD: chronic obstructive pulmonary disease
CNS: central nervous system
CS: chondroitin sulfate
CSF: cerebrospinal fluid
ECM: extracellular matrix
ESCC: esophageal squamous cell carcinoma
GAG: glycosaminoglycan
GVHD: graft‐versus‐host disease
HCC: hepatocellular carcinoma
HF: heart failure
HS: heparan sulfate
HSPG: heparan sulfate proteoglycan
IGD: interglobular domain
IIP: idiopathic interstitial pneumonia
IPF: idiopathic pulmonary fibrosis
iTTP: immune‐mediated thrombotic thrombocytopenic purpura
LC‐MS: liquid chromatography‐mass spectrometry
MM: multiple myeloma
MMP: matrix metalloproteinase
NAFLD: nonalcoholic fatty liver disease
NASH: nonalcoholic steatohepatitis
NSCLC: non‐small cell lung cancer
OA: osteoarthritis
RA: rheumatoid arthritis
SF: synovial fluid
SLE: systemic lupus erythematosus
SLRP: small leucine‐rich proteoglycan
STEMI: ST‐segment elevation myocardial infarction
TAA: thoracic aortic aneurysm
TBI: traumatic brain injury
TLR: Toll‐like receptors
TNF‐α: tumor necrosis factor
UC: ulcerative colitis

## 1 Introduction

**Figure 1:**
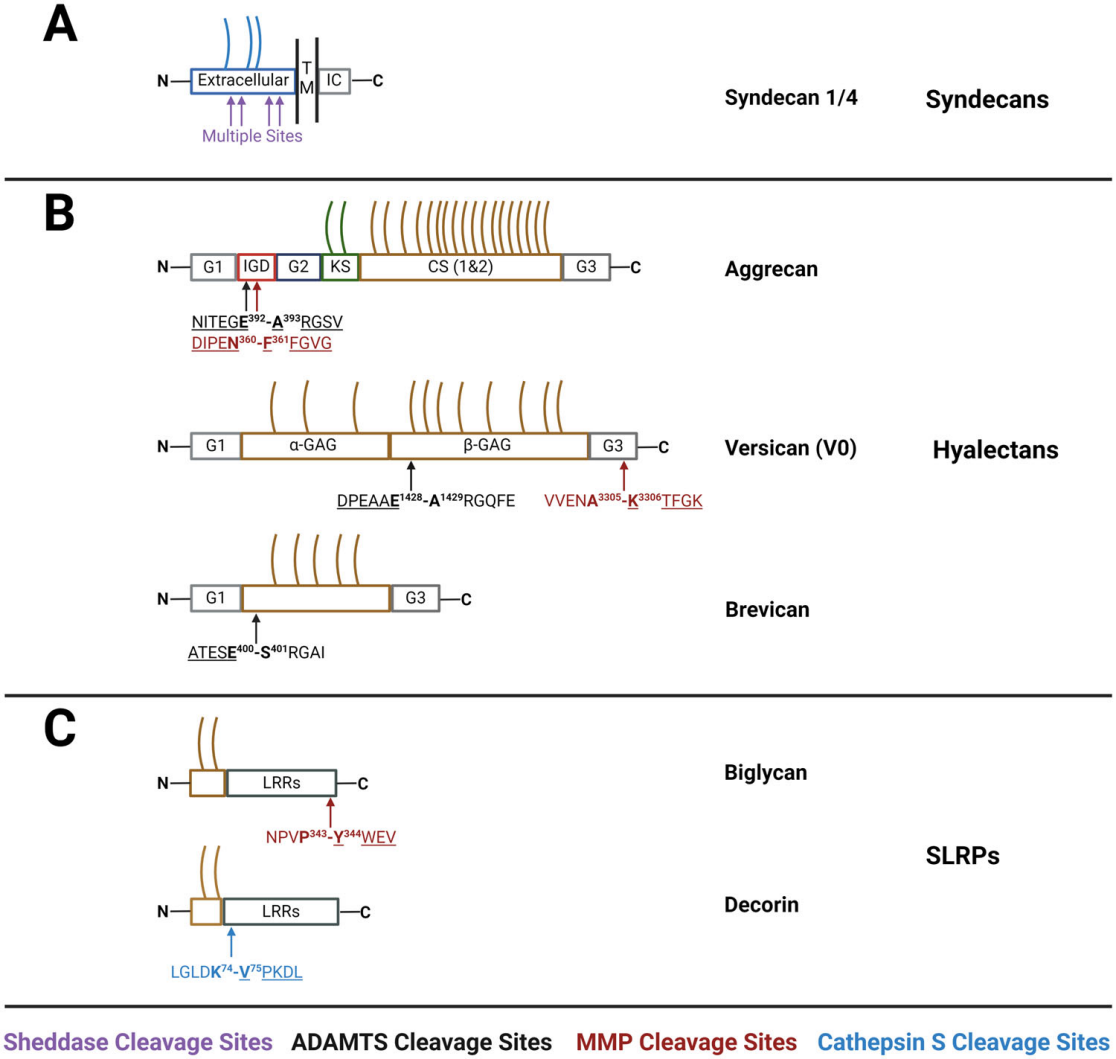
The domain organization and major cleavage sites of proteoglycans discussed in this review. (A) The domain architecture of heparan sulfate proteoglycans syndecans includes extracellular (EC), transmembrane (TM), and intracellular (IC) regions. Please note that syndecan‐1 has additional CS‐GAGs not shown in the figure. (B) Hyalectans are composed of terminal globular domains (G1 and G3 at the N‐ and C‐termini, respectively) and central GAG‐rich regions. (C) Small leucine‐rich proteoglycans (SLRPs) contain a variable number of leucine‐rich repeats (LRR). Neoepitope sequences are underlined. HS‐GAG chains are shown in blue, keratan‐sulfate (KS) GAGs in green, and CS‐GAGs in gold. ADAMTS, a disintegrin and metalloproteinase with thrombospondin motifs; IGD, interglobular domain; KS, keratan sulfate; MMP, matrix metalloproteinase. Figure created with BioRender.

Proteoglycans are complex molecules composed of a protein core and long chains of repeating carbohydrate units called glycosaminoglycans (GAGs). GAGs attract cations from the interstitial fluid to maintain electroneutrality, hence their ability to exert a Gibbs–Donnan osmotic pressure on the tissue and maintain its hydration [1]. Through GAGs, proteoglycans bind a variety of growth factors, cytokines, chemokines, and pathogens [2]. Proteoglycans are classified according to cellular location, protein homology, and the presence of specific modules/domains within their protein cores (Figure 1) [3]. They are crucial components of the extracellular matrix (ECM) and basement membrane [4], but some of them are bound to the cell surface, while few intracellular proteoglycans such as serglycin have been described [5]. Classification into chondroitin sulfate (CS)/dermatan sulfate, and heparan sulfate (HS) proteoglycans is hindered by the ability of certain proteoglycans to carry alternative classes of modifications in different tissues [6]. So far, approximately 90 proteoglycans have been identified and this list is continuously expanding due to advancements in glycoproteomics [5].

In the following sections, we will explore how measuring the levels of soluble proteoglycans and their proteolytic fragments in biological fluids may provide useful biomarkers for pathologies characterized by exacerbated ECM remodeling. We will discuss only studies conducted in human subjects measuring circulating levels of “full‐time” proteoglycans and proteoglycan fragments, with the exclusion of case studies. We identify current bottlenecks in the field and expand on successful examples of proteoglycan‐based biomarkers that can be replicated to overcome the barriers to our ability to diagnose and monitor human diseases.

## 2 Generation of Soluble Proteoglycans: Proteolytic and Non‐Proteolytic Mechanisms

**Figure 2:**
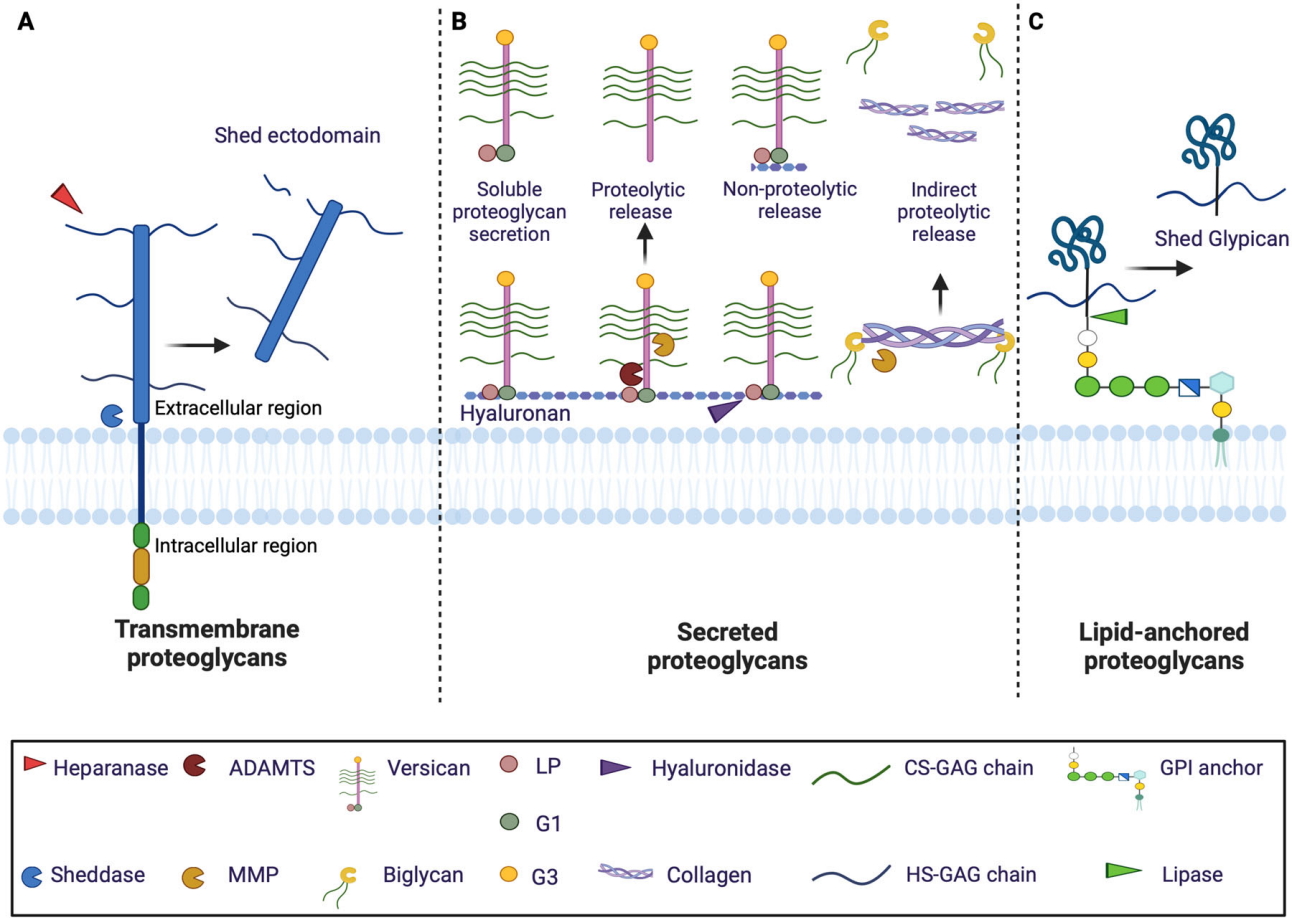
Mechanisms for release of soluble proteoglycans and proteoglycan fragments. (A) Transmembrane proteoglycans such as syndecans are shed from the cell membrane by extracellular and membrane‐bound/transmembrane proteases (‘sheddases’) with the release of the extracellular region (ectodomain). (B) Secreted proteoglycans can be directly released in soluble form by proteolytic and non‐proteolytic mechanism. (C) Lipid‐anchored proteoglycans such as glypicans are shed from the cell membrane by lipases. See text for details. ADAMTS, a disintegrin and metalloproteinase with thrombospondin motifs; CS, chondroitin sulfate; GPI, glycosylphosphatidylinositol; HS, heparan sulfate; LP, link protein; MMP, matrix metalloproteinase. Figure created with BioRender.

Like other ECM components, proteoglycans may be released from their supramolecular assemblies into soluble form due to both proteolytic and non‐proteolytic activities that may work either independently or in concert. The mechanism of this release, which can be either constitutive or induced by inflammatory stimuli, depends on the cellular localization of the proteoglycan. For transmembrane proteoglycans, such as the HS proteoglycans (HSPGs) syndecans, cleavage of the juxtamembrane region with release of the ectodomain (shedding) is mediated by either secreted or membrane‐bound proteases (sheddases) (Figure 2A) [7, 8]. Ectodomain shedding is a powerful way to abolish intracellular signaling events that determine cell behavior by decreasing the number of receptors available for ligand binding on the cell surface [7]. Although constitutive, this phenomenon increases in response to external stimuli [9]. The shed ectodomains can act as decoy receptors that compete with the intact proteoglycan for binding growth factors and cytokines, thus abolishing intracellular cascades or creating local gradients of growth factors within the extracellular/pericellular microenvironment. Alternatively, shed ectodomains can act as paracrine or autocrine effectors [10]. Enzymatic removal of HS‐GAGs by the endoglucuronidase heparanase increases syndecan‐1 shedding by exposing cryptic sites to proteolytic activity or destabilizing the conformation of its protein core (Figure 2A) [11, 12, 13]. The major proteases responsible for cleaving/shedding proteoglycans are zinc metalloproteinases belonging to the metzincin superfamily, in particular matrix metalloproteinases (MMPs), a disintegrin‐like and metalloproteinases (ADAMs), and a disintegrin‐like and metalloproteinase with thrombospondin motifs (ADAMTSs) [14]. In addition to its effects on ECM integrity, this proteolytic activity uncovers new biological functions of proteoglycans with the generation of soluble bioactive fragments called matrikines that function in a paracrine/endocrine manner [14].

While the action of proteases directly contributes to the release of proteoglycan fragments, indirect proteolytic release may occur when proteolysis does not directly affect the proteoglycans but rather a binding partner, such as collagen types I, II, III, and VI, which bind the small leucine‐rich proteoglycans (SLRPs) biglycan and decorin (Figure 2B) [15].

Non‐proteolytic mechanisms of release are specific for the different classes of proteoglycans. In the case of full‐length hyaluronan‐binding CS proteoglycans (CSPG/hyalectans: aggrecan, versican, neurocan, and brevican) non‐proteolytic release may occur through the action of hyaluronidases responsible for degrading hyaluronan, a large unsulfated GAG (Figure 2B) [16, 17]. Full‐length proteoglycans can be directly secreted in a soluble form, for example, by infiltrating activated macrophages upon inflammation and tissue damage (Figure 2B) [16, 17]. However, only from studies using combinations of antibodies against both termini we can conclude that a certain proteoglycan is detected in its full‐length form (as mass spectrometry requires fragmentation, detection of fragments belonging to both termini is not sufficient to establish that these fragments originate from a single, full‐length molecule).

In the case of glypicans, extracellular lipases may cleave the C‐terminal glycosylphosphatidylinositol linkage anchoring them to the plasma membrane, thus releasing the entire proteoglycan (Figure 2C) [3].

Proteoglycans expressed by cell types directly in contact with biological fluids (e.g., endothelial cells, synoviocytes, or macrophages) may be immediately released in their intact or processed form, while those expressed by cell types not directly in contact with biological fluids (e.g., fibroblasts, chondrocytes, smooth muscle cells, neurons, and glial cells) may be released during localized events of tissue damage. Once detached from supramolecular complexes, cleaved/shed, or secreted, proteoglycans and their fragments can either accumulate locally or diffuse into biological fluids depending on the site of origin where they can be further degraded and excreted, or, alternatively, internalized through multiple routes such as clathrin‐ or caveolin‐mediated uptake and micropinocytosis [18]. Although proteoglycans are presumably released with their GAGs still attached, very few studies have experimentally demonstrated the presence of GAGs on soluble proteoglycans in biological fluids [19].

## 3 Advantages and Limitations of Proteoglycans as Biomarkers

A biomarker is defined as “a characteristic that is measured objectively and evaluated as an indicator of normal biological processes, pathogenic processes, or pharmacologic responses to a therapeutic intervention” [20]. To be used in clinical practice, a biomarker must display a series of properties, such as high sensitivity (the ability to detect a disease in patients in whom the disease is truly present), high specificity (the ability to rule out the disease in patients in whom the disease is truly absent), availability, and non‐invasiveness. Biomarkers can be diagnostic (if they stratify risk), prognostic (if they track clinical progression), or theragnostic (if used to measure responsiveness to treatment). These properties are generally defined above or below a certain concentration of the biomarker (the “cut‐off” value).

Proteomics, in particular liquid chromatography‐mass spectrometry (LC‐MS), is widely used for biomarker discovery and validation due to its high sensitivity, high accuracy, and amenability for high‐throughput measurements [21]. However, so far, its application to clinical practice has been limited. Plasma, serum, urine, bronchoalveolar lavage fluid, cerebrospinal fluid (CSF), and synovial fluid (SF) have been extensively used for biomarker discovery and validation. Urine presents particular advantages as a source of biomarkers, such as a low‐complexity proteome and noninvasive collection. The levels of ECM components in the SF, a dialysate of plasma present in the articular joints, are known to correlate with the pathogenetic processes occurring within the cartilage, making it an ideal source of cartilage degradation biomarkers [22]. CSF is quite limited in application due to the invasive procedure required for its sampling, although it provides high diagnostic accuracy for central nervous system (CNS) disorders [23].

As biomarkers, proteoglycans present distinct advantages and challenges. Compared to their proteolytic regulators, proteoglycans are more highly expressed at the protein level, thus allowing for higher sensitivity. Additionally, in plasma most proteases circulate in complex with α2 macroglobulin [24], which may mask crucial epitopes and hinder their detection. On the other hand, the presence of GAGs has hampered the development of immunological and proteomics techniques to measure proteoglycan levels, particularly in the case of the hyalectans, which have a > 300 kDa protein core covalently linked to 20‐100 CS‐GAGs for a total molecular weight of several Da. These disadvantages are circumvented by the development of neoepitope antibodies. Neoepitope antibodies specifically recognize the newly generated N‐ or C‐termini of a proteolytic cleavage product without cross‐reacting with the same epitope when this is presented in the context of the intact protein [25, 26]. As such, neoepitope antibodies not only reflect distinct proteolytic activities but also detect cleavage fragments that are more stable than their corresponding full‐length proteoglycans [25, 26]. The choice of specific neopeptides for neoepitope antibody development is not a streamlined process, as it rarely relies on peptide identification and abundance obtained from proteomic studies of biological samples and it is often based on the identification (by N‐terminal sequencing or LC‐MS) of cleavage fragments generated in vitro. Table 1 provides an overview of proteoglycans investigated as potential biomarkers across a spectrum of pathologies and conditions.

**Table 1:** Specificity and selectivity of selected proteoglycans as biomarkers of pathologic ECM remodeling. Only studies providing cut‐off, sensitivity, and specificity values as estimated by receiver‐operator characteristics (ROC) curve analysis are included.

Proteoglycan (UniProt ID)	Epitope	Condition	Biological fluid	Cut‐off value	Sensitivity (%)	Specificity (%)	References
Aggrecan (P16112)	^393^ARGSV^397^ a	OA	Synovial fluid	> 1 pmol/mL	67	92	[27]
Aggrecan (P16112)	Unknown	Acute type A aortic dissection	Plasma	> 14 ng/mL	97	81	[28]
Biglycan (P21810)	Unknown	NASH	Serum	> 90 pg/mL	94	87	[29]
Brevican (Q96GW7)	^23^DVLEGDSSED^32^ ^401^SRGAIYSIPI^410^ a	AD	Serum	> 9.6 ng/mL	76	75	[30]
Brevican (Q96GW7)	Unknown	AD	Serum	> 2.0 ng/mL	54	67	[31]
Decorin (P07585)	Unknown	AIS	Serum	< 8500 pg/mL	79	63	[32]
Decorin (P07585)^b^	Unknown	ESCC	Plasma	—	74	90	[33]
Decorin (P07585)	^75^VPKDLPPDTT^84^ a	IPF	Serum	> 22 ng/mL	90	100	[34]
Decorin (P07585)	^75^VPKDLPPDTT^84^ a	NSCLC	Serum	> 8.9 ng/mL	63	83	[34]
Endocan (Q9NQ30)	Unknown	CAD	Serum	> 1.7 ng/mL	72	66	[35]
Endocan (Q9NQ30)	Unknown	Slow coronary flow	Serum	> 2.3 ng/mL	77	75	[36]
Endocan (Q9NQ30)	Unknown	AIS	Serum	> 240–270 pg/mL	78	52/63	[37]
Endocan (Q9NQ30)	Unknown	Bacterial infections	Serum	> 2.1 ng/mL	76	85	[38]
Endocan (Q9NQ30)	Unknown	NAFLD	Serum	> 123 pg/mL	72	90	[39]
Endocan (Q9NQ30)	Unknown	STEMI	Serum	> 1.7 ng/mL	76	73	[40]
Endostatin (P39060)	Unknown	COVID‐19	Plasma	> 46 ng/mL	92	71	[41]
Endostatin (P39060)	Unknown	CRC	Serum	> 170 ng/mL	80	66	[42]
Endostatin (P39060)	Unknown	AIS	Plasma	> 190 ng/mL	50	90	[43]
Endostatin (P39060)	Unknown	HF	Serum	> 25 ng/mL	86	67	[44]
Glypican‐3 (P51654)	25Q‐R358	HCC	Serum	> 2 ng/mL	51	90	[45]
Glypican‐4 (O75487)	Unknown	MACE	Serum	> 7.2 ng/mL	39	80	[46]
Syndecan‐1^c^ (P18827)	Unknown	GVHD	Serum	Syndecan‐1 > 160 ng/mL; sIL‐2R > 10,000 pg/mL	82	89	[47]
Syndecan‐1 (P18827)	Unknown	Immunoglobulin A vasculitis	Serum	> 66 ng/mL	67	95	[48]
Syndecan‐1 (P18827)	Unknown	Trauma endotheliopathy	Plasma	≥ 40 ng/mL	62	73	[49]
Syndecan‐1 (P18827)	Unknown	TBI	Plasma	≥ 3 ng/mL	55	52	[50]
Syndecan‐1 (P18827)	Unknown	UC	Serum	> 37 ng/mL	78	77	[51]
Versican (P13611)	Unknown	NSCLC	Plasma	> 1284 pg/mL	70	69	[52]

Abbreviations: AD, Alzheimer's disease; AIS, acute ischemic stroke; CAD, coronary artery disease; CRC, colorectal cancer; CV, cardiovascular; ESCC, esophageal squamous cell carcinoma; GVHD, graft‐versus‐host disease; HCC, hepatocellular carcinoma; HF, heart failure; IPF, idiopathic pulmonary fibrosis; MACE, major adverse cardiovascular events; NAFLD, nonalcoholic fatty liver disease; NASH, nonalcoholic steatohepatitis; NSCLC, non‐small cell lung cancer; OA, osteoarthritis; sIL‐2R, soluble interleukin‐2 receptor; STEMI, ST‐segment elevation myocardial infarction; TBI, traumatic brain injury; UC, ulcerative colitis.

^a^ Neoepitope antibodies.

^b^ In combination smoking, alcohol, and drug consumption.

^c^ In combination with sIL2‐R.

## 4 Proteoglycans and Proteoglycan Cleavage Fragments as Biomarkers of Cartilage Degradation

Aggrecan is responsible for the viscoelastic properties of articular cartilage and its degradation is a major hallmark of degenerative joint diseases such as osteoarthritis (OA) and rheumatoid arthritis (RA) [53]. MMPs and ADAMTSs cleave aggrecan at distinct sites in the interglobular domain (IGD) (Figure 1): Asn360‐Phe361 [54, 55] and Glu392‐Ala393 [56, 57], respectively (UniProt ID: P16112‐1). ADAMTS4 and ADAMTS5 cleave aggrecan at additional sites in the C‐terminal CS‐rich regions with much‐favored kinetics over cleavages in the IGD [26, 58, 59, 60]. However, cleavage in the IGD releases a larger portion of aggrecan from the ECM and is therefore potentially more disruptive to cartilage integrity [61]. Neoepitope antibodies have been developed against cleavage fragments generated by MMPs (anti‐DIPEN and anti‐FFGVG) [55, 62] and ADAMTSs (anti‐ARGSV and anti‐NITEGE) in the IGD, as well as other ADAMTS‐generated fragments in the CS‐rich domains [58, 62, 63]. A 32‐amino acid cleavage fragment generated by dual MMP/ADAMTS activity in the IGD acts in vitro as a pro‐catabolic, anti‐anabolic, and inflammatory matrikine [64].

Since ADAMTS aggrecanase activity precedes that of MMPs in murine models of OA [65], biomarker studies have been focusing on the ADAMTS‐generated aggrecan ARGSV neoepitope, for which specific LC‐MS/MS [66] and ELISA [27, 67, 68, 69, 70, 71] methods have been developed for detection in SF, urine, and plasma/serum. Aggrecan ARGSV neopeptides were increased in RA, OA, and acute knee injury patients (Table 1) [27, 67, 68, 69, 70, 71]. Serum aggrecan ARGSV neoepitope levels positively correlated with age and Western Ontario and McMaster Universities Osteoarthritis Index score [69], while no correlation was found with the Kellgren–Lawrence disease score, pain score or structural outcomes (minimum joint space, osteophyte area, and subchondral bone density) [71]. Following surgery, serum ARGSV levels did not significantly differ from control subjects [67]. In agreement with this, low levels of ARGSV in SF and plasma were associated with the progression of radiographic knee OA which was interpreted as a lack of aggrecan biosynthesis in the joints [67, 70]. A lack of correlation between aggrecan ARGSV levels in serum and SF, with higher levels in the latter, indicates that this neoepitope reflects local pathological processes in the joint, suggesting that the SF may be a better choice as a source of this candidate biomarker [71]. In a phase II clinical trial, patients receiving a 300 mg dose of an ADAMTS5 inhibitor had a 58.5% reduction in their serum aggrecan ARGSV‐neopeptides after a 4‐week treatment, but these were not associated with preservation of cartilage thickness as measured by magnetic resonance imaging or pain score [72]. In a phase I clinical trial, changes in serum aggrecan ARGSV levels were observed only in the treated group, not in the placebo group [73].

Compared to aggrecan, other proteoglycans have been poorly investigated as biomarkers of degenerative cartilage diseases. Among the SLRPs, biglycan levels were increased in the SF of patients with advanced OA and RA compared to patients with meniscus tears, while no differences were observed for soluble decorin [74]. A few studies have also investigated syndecans in the context of OA/RA. Serum syndecan‐1 levels were increased in RA patients and decreased following a 6 week‐anti‐inflammatory treatment with methotrexate or tumor necrosis factor (TNF)‐α inhibitors [75]. Serum syndecan‐4 levels were increased in RA patients compared to OA patients or healthy controls, while the SF showed similar levels in RA and OA patients, suggesting a contribution from systemic glycocalyx shedding (see Section 7). In both SF and serum of RA patients, syndecan‐4 levels correlated with disease severity score [76].

## 5 Proteoglycans and Proteoglycan Cleavage Fragments as Potential Biomarkers in Cardiovascular Pathologies

Versican is the most abundant proteoglycan in the vasculature and heart, where its expression is essential for normal morphogenesis [77]. Immunohistochemical analysis using human tissues has shown that versican expression dramatically increases in thoracic aortic aneurysmal (TAA) lesions [78], atherosclerotic plaques, restenosis [79], and cardiac fibrosis [80]. Accumulation of versican and other proteoglycans is known to contribute to the pathological events leading to atherosclerotic plaque deposition since the GAGs have a high capacity for binding and retaining low‐density lipoproteins [79].

High molecular weight versican fragments have been detected in plasma by immunoblot using antibodies against the G3 domain [81, 82]. However, due to the limited availability of specific, sensitive anti‐versican antibodies, few studies have measured soluble versican levels. In a notable exception, a versican neopeptide generated by cleavage of MMP8 and MMP12 at Ala3305‐Lys3306 (UniProt ID: P13611‐1) in the G3 domain (Figure 1), and named VCANM, was used to raise neoepitope antibodies which were selected for ELISA development [83]. VCANM was initially identified by LC‐MS/MS following in vitro digestion of a chicken proteoglycan extract and selected for its complete conservation across different species. Plasma levels of VCANM were increased in atherosclerotic patients compared to non‐atherosclerotic controls [83], possibly reflecting total versican levels. This study exemplifies the power of an approach that starts from in vitro neopeptide discovery and characterization. Furthermore, the ability to link a neopeptide to a specific protease or a protease family makes VCANM and similar neopeptides excellent tools to monitor proteoglycanolytic activities in vivo.

Although traditionally investigated as a cartilage proteoglycan, aggrecan is also expressed in a number of vascular beds [84]. Similar to versican, investigation of aggrecan as a vascular biomarker has been extremely limited. A notable exception is the study by König et al. [28] showing that aggrecan plasma levels were significantly increased in patients with acute type A aortic dissection compared to patients with TAA, myocardial infarction or healthy volunteers. These findings partially corroborated a previous study that analyzed TAA tissues by immunohistochemistry but did not differentiate between TAA and dissected lesions [78]. As a biomarker, aggrecan was able to detect an acute type A aortic dissection with a sensitivity of 97% and specificity of 81% (Table 1) [28]. The study used a commercially available sandwich ELISA kit for which no information on the epitope is available.

Compared to aggrecan and versican, numerous studies have investigated HSPGs and SLRPs as biomarkers of cardiovascular pathologies [3, 10, 15]. We will summarize these studies here, grouping the different proteoglycans according to disease.

Patients with hypertension exhibited elevated serum levels of endostatin [85], endocan [86, 87, 88], and osteoglycin [89]. Endostatin is an antiangiogenic C‐terminal 22 kDa fragment generated by cleavage of the basement membrane HSPG collagen XVIII by MMPs and cathepsins [4]. Higher serum endostatin levels were associated with indices of hypertensive target‐organ damage such as endothelium‐dependent vasodilation, left ventricular mass, and urinary albumin/creatinine ratio in two community‐based cohorts of elderly participants [85]. Ostoglycin and endocan levels correlated with arterial stiffness [87, 89]. If further validated, these studies may indicate that endocan and osteoglycin represent candidate biomarkers of arterial stiffness, which is currently evaluated by ultrasound imaging.

Chronic stable angina pectoris was also associated with higher plasma/serum levels of endocan and osteoglycin. Plasma osteoglycin was associated with the probability of a major adverse cardiovascular event in patients who underwent coronary angiography for acute coronary syndrome or stable angina pectoris [90]. In patients with chronic stable angina, serum endocan levels positively correlated with high‐sensitivity C‐reactive protein [35]. In patients with coronary artery disease (CAD), coronary collateral circulation (CCC) is an adaptive response to chronic myocardial ischemia which protects the heart from tissue damage and prevents infarction by diverting blood flow to collateral vessels [91]. CAD patients with good CCC values (Cohen–Rentrop grades 2–3) had lower endocan levels compared to those with poor CCC (Cohen–Rentrop grade 0–1) (Table 1) [35]. Increased serum glypican‐4 levels were also associated with an increased risk of adverse cardiovascular events in patients who underwent coronary angiography [46].

Alongside the few studies that have measured circulating levels of hyalectans as biomarkers of atherosclerosis, serum endocan has recently emerged as an alternative potential biomarker, with a distinct ability to detect disease before clinical onset. In type 2 diabetic patients, high serum endocan levels correlated with a pro‐atherosclerotic lipoprotein profile and markers of subclinical atherosclerosis such as carotid intima‐media thickness [92, 93]. Compared to healthy controls, obese children had higher serum endocan levels which correlated with carotid intima‐media thickness, a marker of preclinical atherosclerosis [94]. In symptomatic atherosclerotic patients, serum/plasma endocan levels correlated with higher coronary atherosclerosis scores [95, 96]. Patients with heart failure (HF) showed significantly increased plasma/serum levels of osteoglycin [97], glypican‐4 [98], endocan [99], syndecan‐1 [100, 101], syndecan‐4 [102, 103, 104], endostatin [44], and perlecan [105], compared with control subjects. Higher plasma osteoglycin levels were associated with significantly increased 18‐month mortality in HF patients and correlated negatively with left ventricular volume and positively with fibrotic markers such as interleukin‐6 and the N‐propeptide of procollagen type I [97]. Serum glypican‐4 predicted mortality in a large cohort of HF patients in a 2‐year follow‐up [98]. Plasma endocan was an independent prognostic factor of HF [99]. Serum syndecan‐1 levels were a predictor of disease progression and mortality [101]. The inclusion of serum syndecan‐1 in a panel of biomarkers comprising the N‐terminal probrain‐type natriuretic peptide (NT‐proBNP) or troponin further strengthened the panels' prognostic value [101]. In patients with chronic HF, higher plasma syndecan‐1 levels displayed higher NT‐proBNP and positively correlated with markers of cardiac fibrosis such as galectin‐3, periostin, and ST‐2, but not inflammation [101]. In HF patients with preserved ejection fraction, plasma syndecan‐1 was associated with an increased risk of mortality or rehospitalization [100]. Increased serum syndecan‐4 levels in HF patients correlated with left ventricular ejection fraction, suggesting a potential application for syndecan‐4 as a biomarker of left ventricular remodeling [102, 103]. In patients with chronic HF, higher serum endostatin levels positively correlated with NT‐proBNP concentrations and were associated with high‐risk mortality over a 31‐month follow‐up period (Table 1) [44]. Plasma perlecan levels were associated with an increased risk of hospitalization for decompensated HF and cardiac mortality [105].

Increased levels of circulating syndecan‐1 [97], endocan [36, 37, 106], and endostatin [107] are associated with the occurrence of ischemic events, in particular acute ischemic stroke (AIS). In a small cohort of patients undergoing surgery on the ascending aorta for cardiopulmonary bypass or infrarenal aortic aneurysms, events of global or regional ischemia were associated with a drastic increase in plasma syndecan‐1 levels (42‐ and 65‐fold, respectively) [108]. Serum endocan levels were higher in patients with slow coronary flow, a cause of ischemia, than in controls [106]. Serum endocan levels correlated with high levels of highly sensitive C‐reactive protein and, at a cut‐off of 2.3 ng/mL, predicted slow coronary flow with a sensitivity of 77% and specificity of 75% [36]. Patients with AIS had higher levels of serum endocan which predicted an unfavorable outcome (Table 1) [37]. Increased plasma endostatin levels in AIS patients were associated with risk of long‐term mortality, severe disability [107, 109, 110], and post‐stroke cognitive impairment [111]. Endostatin levels higher than 185 ng/mL independently predicted the occurrence of ischemic events in patients with symptomatic intracranial stenosis, although with limited sensitivity (Table 1) [43].

AIS patients experienced a gradual decrease in plasma decorin levels within the first 2 weeks from the onset of the event, which was more dramatic in those with poor outcomes than in those with favorable outcomes [112]. Plasma decorin levels positively correlated with those of MMP2, which were similarly decreased in AIS patients compared to non‐AIS controls [32].

As a general marker of endothelial damage, circulating syndecan‐1 levels are elevated in several cardiovascular conditions. Plasma syndecan‐1 levels were increased in trauma patients compared to healthy controls [49, 113, 114]. At a cut‐off of ≥ 40 ng/mL, plasma syndecan‐1 identified 34% of patients with trauma endotheliopathy with a sensitivity of 62% and a specificity of 73%. Moreover, plasma syndecan‐1 levels were significantly associated with an increased 30‐day in‐hospital mortality [49]. Higher plasma syndecan‐1 levels in trauma patients were associated with pro‐inflammatory cytokines and markers of endothelial damage, coagulation, and fibrinolysis [115, 116, 117, 118, 119, 120]. Plasma syndecan‐1 levels also increased in hospitalized patients recovering from major trauma who developed venous thromboembolism [121], acute pulmonary embolism [122], or reduced plasma colloid osmotic pressure [123]. When fresh frozen plasma was administered to severely injured patients in hemorrhagic shock [124] or to coagulopathic non‐bleeding critically ill patients before an invasive procedure [125], plasma syndecan‐1 levels decreased, suggesting that the procedure effectively preserved the endothelial glycocalyx. These studies indicate that high plasma levels of syndecan‐1 are indicative of endothelium injury in trauma.

Patients with ST‐segment elevation myocardial infarction (STEMI) had higher levels of both syndecan‐1 [126] and endocan [40, 127, 128, 129]. Plasma syndecan‐1 levels were independently associated with 6‐month mortality after STEMI [126]. In STEMI patients, higher serum endocan levels were associated with major adverse cardiac effects [127, 128, 129]. Increased serum endostatin levels have been also associated with an increased risk of myocardial infarction, but analysis in large community‐based cohorts did not support this finding after correction for C‐reactive protein [130]. High serum endostatin was associated with a higher risk of all‐cause mortality in a randomized, placebo‐controlled trial of stable coronary heart disease patients evaluating 14‐day treatment with clarithromycin to eradicate *Chlamydia pneumoniae* infections, suggesting that a dysregulated antiangiogenic response may trigger acute events in atherosclerotic subjects [131]. Serum syndecan‐1 levels were also found to be increased in patients with Kawasaki disease [132, 133], Takotsubo cardiomyopathy [134], Clarkson disease [135], and immune‐mediated thrombotic thrombocytopenic purpura (iTTP) [136]. Compared to healthy controls, patients with acute iTTP had higher plasma syndecan‐1 levels at admission which predicted in‐hospital mortality and recurrence [137].

Pre‐eclampsia is the only cardiovascular disease where circulating levels of syndecan‐1 [138, 139, 140, 141] and perlecan [142, 143] decreased compared to healthy or preeclamptic pregnant women, likely as a result of transcriptional downregulation in the placenta [144].

## 6 Proteoglycans and Proteoglycan Cleavage Fragments as Potential Biomarkers in Kidney Disease

In the pathologies of the kidney, soluble levels of endothelial damage markers such as the HSPGs syndecan‐1 and ‐4 [145], the collagen XVIII fragment endostatin [146], SLRPs such as biglycan [15], as well as versican fragments [147] were found to be increased. Compared to healthy controls, patients with chronic kidney disease (CKD) had higher plasma syndecan‐1 levels [145]. Patients with acute decompensated HF who experienced acute kidney injury as a complication had higher syndecan‐1 plasma levels compared to patients who had stable CKD and these were associated with longer hospitalization and higher mortality rates [148]. In pediatric patients subjected to cardiac surgery, higher urine [149] and plasma [150] syndecan‐1 levels were associated with severe acute kidney injury. These findings were independently replicated in an adult cohort using plasma/serum syndecan‐1 [151, 152].

Elevated plasma endostatin levels were strongly and independently associated with disease progression and cardiovascular events in CKD patients [153, 154]. In CKD patients receiving kidney transplants, elevated serum endostatin levels were associated with graft loss [155].

High serum syndecan‐4 levels were associated with increased mortality in hemodialysis patients [156]. In contrast, hemodialysis patients with plasma VCANM concentrations in the lowest quartile had a survival of 152 days compared to 1295 days for patients with plasma VCANM in the highest quartile [147], suggesting an association between decreased versican proteolysis/versican accumulation and mortality. In a large cohort (372 subjects) of patients undergoing hemodialysis, elevated plasma endostatin levels ( > 200 ng/mL) were independently associated with an increased risk of cardiovascular events such as acute coronary artery syndrome, heart failure, ventricular arrhythmia, cardiac arrest, and sudden death [157].

Plasma biglycan levels increased in patients with diabetic nephropathy, lupus nephritis, and in those with acute renal allograft rejection, and correlated with increased levels of the pro‐inflammatory chemokines/cytokines RANTES, IL‐1β, TNF‐α, MCP‐1 and CXCL13 [158]. Urine decorin levels also increased in a small cohort of nephropathic patients with primary glomerular disease where they negatively correlated with creatinine clearance [159]. A combined approach using multi‐lectin affinity chromatography followed by LC‐MS/MS identified lumican as one of 13 upregulated glycoproteins in the plasma of diabetic nephropathy patients compared to type 2 diabetic patients without nephropathy. However, it is important to highlight that the two cohorts only included 6 patients each (with no healthy controls) and the proteome analysis was semi‐quantitative [160]. Increased serum endostatin was identified as a predictor of progression to end‐stage kidney disease in a very large cohort (754 individuals) of patients with type 1 and type 2 diabetes using the SOMAscan platform [161], thus confirming previous associations with diabetic kidney disease severity and mortality in patients with type 2 diabetes [162, 163, 164]. A recent meta‐analysis found a significant association between high serum endocan levels, type 2 diabetes, and diabetic complications [165].

Levels of the C‐terminal fragment of perlecan, endorepellin, were elevated in the urine of patients with chronic allograft nephropathy [166] and in the serum of patients with acute aortic allograft rejection [167] compared to healthy volunteers.

## 7 Proteoglycans and Proteoglycan Cleavage Fragments in Infectious Diseases

Inflammation involves an immune response to exogenous or endogenous stimuli that, if dysregulated, may lead to a variety of pathologies. By their ability to bind to a number of growth factors, chemokines, cytokines, and proteases, proteoglycans act as important modulators of the immune response [168, 169]. Infections by viral or bacterial pathogens trigger a robust remodeling of the host ECM basement membrane and glycocalyx by activated white blood cells, resulting in the release of proteoglycans and their fragments into the circulation [16, 17].

Elevated levels of circulating syndecans, derived from exacerbated endothelial shedding, indicate glycocalyx damage, characterized by increased leukocyte recruitment, vascular permeability, and intravascular coagulation. The main proteases responsible for syndecan shedding are plasmin [10], thrombin [10], MMP7 [170], MMP9 [171, 172], and ADAM17 [173]. Shed syndecan‐4 has been proposed as a marker of acute pneumonia since serum syndecan‐4 levels were significantly higher in patients with acute pneumonia compared to healthy volunteers and negatively correlated with pneumonia severity score [174, 175].

Increased syndecan‐1 shedding is induced by viral infections, such as dengue [176] and SARS‐CoV‐2 [177]. In dengue patients, increased plasma syndecan‐1 levels were found to be associated with severe plasma leakage [178, 179], which may be explained by the ability of shed syndecan‐1 to promote microvascular permeability [176]. Increased serum syndecan‐1 levels correlated with COVID‐19 infection severity [180, 181, 182, 183, 184, 185] and d‐dimer [184, 185], a hypercoagulation marker. Plasma syndecan‐1 levels were also higher in patients who did not survive COVID‐19 infection [186, 187, 188], suggesting an association with mortality. A recent systematic review and meta‐analysis confirmed the association between elevated shed syndecan‐1 levels and poor outcomes in COVID‐19 patients [177]. Another proteoglycan potentially involved in COVID‐19 may be collagen XVIII, as plasma endostatin levels higher than 46 ng/mL were associated with an increased risk of death in patients admitted to the intensive care unit (Table 1) [41].

Other than syndecans, an important role in inflammatory diseases is exerted by the hyalectans. The interaction between versican and hyaluronan increases the viscosity of the ECM and inhibits the migration of CD4^+^ and CD8^+^ T cells, thus acting as an immunological barrier during viral infections [189] and cancer [190] (see Section 11). The size of versican and the number of its GAGs have limited the development of immunological techniques able to detect soluble versican in biological fluids. Versican cleavage fragments may offer exciting opportunities instead. We mentioned VCANM, the fragment generated by MMP8/12 activity [83], above, but so far, VCANM levels have not been measured in infectious diseases. The best‐characterized cleavage site in versican is by ADAMTS proteases at a Glu‐Ala bond in the GAGβ domain (Glu441‐Ala442 in V1, Glu1428‐Ala1429 in V0) [191], although other cleavage sites have been reported [192]. This proteolytic event releases a bioactive 70kDa‐fragment containing the N‐terminal G1 domain and the C‐terminal DPEAAE neoepitope called versikine, which has been implicated in immunological signaling and apoptosis [193, 194, 195]. However, similar to VCANM, so far versikine levels have not been measured in patients with infectious diseases, even though a specific sandwich ELISA has been recently described [196].

Septic patients had high circulating levels of decorin [197], syndecan‐1 [198], and glypican‐1, ‐3, and ‐4 [199], compared to healthy volunteers, correlating with markers of disease severity. High syndecan‐1 plasma levels correlated with the need for mechanical ventilation in septic patients with pneumonia, while endocan levels decreased over time following admission to the emergency department [199]. Plasma/serum syndecan‐1 levels were increased in septic shock patients admitted to the intensive care unit compared to matched controls, and correlated with disseminated intravascular coagulation, multiorgan failure, thrombocytopenia, and lethal outcome [200, 201, 202, 203, 204, 205, 206, 207].

High serum endocan levels are an independent predictor of bacterial infections in cirrhotic patients (Table 1) [38, 208].

## 8 Proteoglycans and Proteoglycan Cleavage Fragments in Fibrotic Lung Disorders

In contrast to infectious disease, circulating levels of syndecans [209] and the MMP‐generated versican fragment VCANM [210, 211] were found to be decreased in patients with fibrotic lung disorders, while levels of SLRPs significantly varied depending on the etiology [34, 212]. Patients with acute idiopathic pulmonary fibrosis (IPF) had lower serum syndecan‐4 levels than clinically stable patients. The prognosis was significantly worse in patients with high baseline serum syndecan‐4 levels than in those with low baseline levels [209]. In contrast, levels of both full‐length decorin and cathepsin S‐generated decorin fragments were increased in these patients compared to non‐fibrotic controls (Table 1) [34, 212]. Biglycan fragments generated by MMP2 or MMP9 were higher in patients with progressive IPF compared to those with stable disease and were associated with poor survival [213].

VCANM levels were lower in patients with acute idiopathic interstitial pneumonia (IIP) compared to stable patients and were associated with an increased mortality risk and shorter survival time [210]. Similarly, IIP patients had lower serum decorin levels than patients with stable disease or healthy controls [212].

Serum levels of VCANM were also found to decrease in patients with exacerbated chronic obstructive pulmonary disease (COPD) compared to those in the recovery phase [211], when total versican levels in the lung are known to rise [214], suggesting that versican accumulation may be driven by reduced proteolysis by MMPs.

These findings indicate that dysregulated turnover of versican and decorin alters levels of these proteoglycans in the lung ECM, a process that may be partially responsible for fibrotic manifestations of IPF, IIP, and COPD.

Higher serum endostatin levels were significantly associated with disease severity, worse hemodynamics, and mortality in patients with pulmonary arterial hypertension, possibly via its inhibitory effect on vascular growth [215, 216, 217].

## 9 Proteoglycans and Proteoglycan Cleavage Fragments in Liver Disease

Serum biglycan levels were higher in chronic hepatitis patients compared to healthy controls and positively correlated with fibrosis and necroinflammatory activity [218]. Patients with nonalcoholic steatohepatitis (NASH) had higher circulating levels of serum biglycan levels than controls which correlated with fibrosis score [29]. Patients with NASH and those with nonalcoholic fatty liver disease (NAFLD) had increased serum endocan levels [219]. In contrast, Tok et al. [220] and Erman et al. [39] found that patients with NAFLD had lower serum endocan levels compared to healthy controls (Table 1).

## 10 Proteoglycans and Proteoglycan Cleavage Fragments in Autoimmunity

Autoimmune diseases present many clinical similarities with infectious diseases and cancer, with levels of circulating proteoglycans found to be similarly altered. For example, serum levels of endocan [221] and syndecan‐1 [51] were increased in ulcerative colitis (UC) patients compared to healthy controls. Patients with active (more severe) UC had higher serum syndecan‐1 which positively correlated with disease score (Table 1) [51]. In patients with chronic hepatitis, serum syndecan‐1 levels diagnosed liver fibrosis with a positive predictive value of 82% [222].

Patients with systemic sclerosis had increased serum syndecan‐1 [223] and endostatin levels [224] as well as higher levels of serum biglycan fragments derived from MMP9/MMP12 activity at an early stage compared to the late stage of the disease [225]. A recent analysis in patients with preclinical systemic sclerosis using SomaScan analysis strongly associated serum endostatin levels with disease progression [226].

Serum levels of both syndecan‐1 [227, 228, 229] and endocan [230] were increased in patients with systemic lupus erythematosus (SLE) compared to normal controls. In female SLE patients, higher serum endocan levels positively correlated with markers of subclinical atherosclerosis such as carotid intima‐media thickness, body mass index, and erythrocyte sedimentation rate [231].

Biglycan is a pro‐inflammatory SLRP binding the innate immunity Toll‐like receptors (TLRs) 2 and 4 [3]. Plasma biglycan levels increased fivefold in patients with lupus nephritis compared to healthy controls and were associated with albuminuria and increased levels of chemokine (C‐X‐C motif) ligand (CXCL) 9,10, and 13 [158, 232]. The increased levels of biglycan were most likely due to increased biglycan synthesis rather than proteolytic release from the ECM (Figure 2B), since elevated levels of pro‐inflammatory cytokines/chemokines IL‐1β, TNF‐α, MCP‐1, and RANTES induced the mRNA expression of biglycan by macrophages via TLR2 and TLR4 [158].

Serum syndecan‐1 levels increased in Crohn's disease patients [233], particularly in those with an active pathologic manifestation compared to those in remission [234]. In patients receiving allogeneic stem‐cell transplantation, serum syndecan‐1 levels were elevated in those who developed acute graft‐versus‐host disease (GVHD). The combination of syndecan‐1 and soluble IL‐2 receptor detected GVHD with a sensitivity of 82%, and a specificity of 89% (Table 1) [47]. Serum syndecan‐1 levels above 66 ng/mL identified immunoglobulin‐A vasculitis in pediatric patients with sensitivity and specificity of 67% and 95%, respectively [48]. In pediatric celiac disease patients, serum syndecan‐1 levels were increased compared to children with nonspecific abdominal pain and correlated with disease progression and grade [235]. Serum syndecan‐4 levels were increased in patients with atopic dermatitis and correlated with disease severity [236].

## 11 Proteoglycans and Proteoglycan Cleavage Fragments in Cancer

Soluble syndecan‐1 levels are significantly increased in both liquid and solid tumors. Among the liquid tumors, increased circulating levels of syndecan‐1 have been reported in patients with chronic lymphocytic leukemia [237] and multiple myeloma (MM) [238, 239, 240, 241]. Shed syndecan‐1 promotes the growth of myeloma in vivo, a process enhanced by heparanase through a dual action involving induction of syndecan‐1 synthesis and enzymatic removal of HS‐GAGs from the protein core (Figure 2A) [11]. When both syndecan‐1 and heparanase were highly expressed by myeloma cells, circulating levels of shed syndecan‐1 also increased, a phenomenon that positively correlated with poor prognosis [242] and disease stage [243]. Out of 174 MM patients analyzed for the presence of serum syndecan‐1, 79% had significantly higher levels compared to healthy volunteers, with those in the high‐expression group having lower survival rates compared to patients in the low‐expression group [239]. In a small Greek MM cohort, serum syndecan‐1 levels directly correlated with disease stage and inversely correlated with 5‐year survival (47% in the high serum syndecan‐1 group vs. 100% in patients with normal syndecan‐1 levels) [243]. Chemotherapy responders exhibited a decrease in their serum syndecan‐1 levels compared to non‐responders [243], a finding that was replicated in a Korean cohort [244]. Based on these findings, serum syndecan‐1 has been proposed as an independent prognostic biomarker of MM [239, 243], and indeed the evidence for syndecan‐1 is stronger than decorin [245, 246] or versican [247]. MM patients also had higher bone marrow plasma decorin levels compared to healthy controls which were associated with better responses to treatment in a retrospective study [245], while another study found instead lower levels in the bone marrow plasma of MM patients compared to controls [246]. Versican levels in bone marrow plasma and blood serum of MM patients with stage II and III disease were elevated compared to healthy volunteers or patients with iTTP [247].

Among solid tumors, circulating levels of syndecan‐1 have been found to increase in patients with colorectal [248], lung [249, 250], breast [251], hepatocellular [252], and prostate cancer [253, 254]. Patients with metastatic colorectal cancer had higher plasma levels of syndecan‐1 and glypican‐4, which were associated with poor survival independently of clinical characteristics [248]. Baseline levels of shed syndecan‐1 were lower in the serum of colorectal cancer patients who responded to chemotherapy compared to non‐responders and had higher rates of disease‐free survival, suggesting that serum syndecan‐1 levels may be used to determine the likelihood of patients' response to certain chemotherapeutic treatments and predict tumor relapse [255]. In two independent prospective clinical trials, serum syndecan‐1 and lactate dehydrogenase were able to stratify patients at risk of death for metastatic colorectal cancer [256]. In colorectal cancer, shed syndecan‐1 may have a protumorigenic role mediated by its intact HS‐GAGs which can bind heparin‐binding epithelial growth factor‐like factor (HB‐EGF) on epithelial cells, resulting in the activation/phosphorylation of the EGF receptor, stimulation of the downstream Ras‐Raf‐MEK‐ERK pathway and increased drug resistance [255].

High shed syndecan‐1 levels have been reported in the serum of patients with lung cancer, where they were associated with serum levels of basic fibroblast growth factor and poor clinical outcome [249, 250]. Additionally, patients with lung cancer had higher serum biglycan levels than healthy controls or patients with less invasive adenocarcinomas [257].

Circulating levels of versican [52] and decorin [243] are abundant in patients with non‐small cell lung cancer (NSCLC). Plasma versican levels were higher as NSCLC progressed, correlating with disease stage and lymph node metastasis [52]. At a cut‐off of 1.3 ng/mL, versican diagnosed NSCLC with a sensitivity of 70%, and specificity of 69%. At an optimal cut‐off value of 8.9 ng/mL, cathepsin S‐derived serum decorin fragments detected NSCLC with a sensitivity of 63% and a specificity of 83% (Table 1) [34].

In addition to syndecan‐1 [251], elevated circulating levels of decorin [258] and glypican‐4 [259] were found in breast cancer patients. Elevated plasma levels of decorin were found to be an independent predictive factor for advanced breast cancer [258], while plasma glypican‐4 levels predicted 2‐year survival in metastatic breast cancer patients with an optimal cut‐off of 4.8 ng/mL [259].

In hepatocellular carcinoma (HCC) patients, circulating levels of syndecan‐1 [252], glypican‐3 [45, 252, 260, 261], and endocan [262, 263] were higher than patients with liver fibrosis/cirrhosis, chronic hepatitis or healthy controls. A combination of serum syndecan‐1, glypican 3, and sulfatase 2, an endosulfatase catalyzing the 6‐O‐desulfation of proteoglycan HS‐GAGs and a CSPG itself [264], predicted poor prognosis in HCC patients [252]. Cleavage of glypican by unknown protease(s) at R358‐S359 releases a soluble N‐terminal fragment which seems to be the major fragment released into circulation [45]. In two independent studies, while glypican‐3 had a modest sensitivity (51%–56%) for the diagnosis of HCC, a combination of glypican‐3 and alpha‐fetoprotein increased the sensitivity to 72%–75% [45, 261]. In HCC patients, syndecan‐1 and endocan levels were associated with an increased risk of tumor relapse and death [262]. Higher serum endocan levels were associated with poor liver function, tumor size, and mortality in HCC patients [263].

Serum syndecan‐1 levels also increased in patients with muscle‐invasive bladder cancers compared to noninvasive, low‐grade bladder cancers [264, 266], being independently associated with poor survival [267], and in patients with pancreatic ductal adenocarcinoma [268].

Compared to subjects with the lowest quartile of plasma decorin, those with the highest quartile had a significantly lower risk of having esophageal squamous cell carcinoma (ESCC) [33]. The combination of plasma decorin and three additional risk factors (smoking, alcohol, and drug consumption) correctly classified ~82% of ESCC patients (Table 1) [33]. These findings indicate that the inclusion of plasma decorin in a panel of risk factors may be used to screen patients at risk of developing ESCC.

Multiple perlecan fragments, mostly derived from domain IV, were elevated in the serum of prostate cancer patients compared to control subjects. These perlecan fragments correlated with levels of the perlecan‐degrading protease MMP7 in the tissue, suggesting that they were proteolytically released by MMP7 from the bone marrow and stroma [269].

Elevated serum endostatin levels were found in patients with colorectal cancer [42, 270], nasopharyngeal carcinoma [271], gastric cancer [272], bladder cancer [273], non‐Hodgkin lymphoma [274], non‐small cell lung cancer [275], and pancreatic cancer [276], correlating with disease progression, poor overall survival and poor prognosis. Given the antiangiogenic properties of endostatin, recombinant human endostatin has been tested in clinical trials for the treatment of several solid tumors [146].

As discussed in the following section, brevican expression is abundant in the CNS [3]. Several brevican and versican‐derived O‐linked glycopeptides were detected at differential levels in brain tissue from glioblastoma patients compared to controls using LC‐MS/MS [277]. ADAMTS4 and ADAMTS5, but not ADAMTS1, cleave rat brevican at Glu395‐Ser396 (corresponding to Glu400‐Ser401 in human brevican, UniProt ID Q96GW7) [278], a proteolytic activity that in a 2008 study was linked to glioma invasiveness [279]. These findings suggest that dysregulated proteolysis of CNS proteoglycans, in particular brevican, is involved in the growth and progression of CNS tumors. Quite surprisingly, the investigation of brevican fragments as glioma biomarkers has not been further explored.

## 12 Proteoglycans and Proteoglycan Cleavage Fragments in Central Nervous System Disorders

The primary proteoglycans in the CNS are brevican, neurocan, and versican, specifically its V2 isoform [3]. Proteoglycans and their fragments have been implicated in several neurological diseases and conditions and are detectable in the CSF. The CSF Proteome resource (https://proteomics.uib.no/csf-pr-id/) (accessed 1 May 2024) [280] retrieves multiple brevican and neurocan peptides throughout Alzheimer's disease (AD), multiple sclerosis, Parkinson's disease and amyotrophic lateral sclerosis proteomic studies.

CSF concentrations of brevican and neurocan positively correlated with age and markers of neurodegeneration (total tau, phosphorylated Tau, neurofilament‐L, and Aβ1‐40) and cognitive decline in AD patients [281, 282], although the lack of significant difference in CSF concentrations between AD patients and cognitively normal controls [283, 284, 285] questions their suitability as AD‐specific biomarkers. A specific ELISA for a C‐terminal brevican neoepitope, Brev‐A, generated by ADAMTS4 cleavage at Ser401, was developed, with the neoepitope antibody also detecting brevican fragments in brain tissue [30]. Serum levels of Brev‐A, and its ratio in comparison to N‐terminal fragments (N‐Brev), were significantly increased in patients with dementia compared to AD. At an optimal cut‐off of 9.6 ng/mL, the N‐Brev/Brev‐A ratio discriminated dementia from AD with sensitivity and specificity of 76% and 75%, respectively [30] (Table 1). Both N‐terminal and C‐terminal brevican peptides were noticeably decreased in vascular dementia when compared to both AD and control cohorts [285]. Cranial radiotherapy led to a steady decrease in CSF levels of both brevican and neurocan in patients with brain metastasis due to small‐cell lung cancer, with a strong correlation between brevican levels and the non‐amyloidogenic soluble amyloid precursor protein α (sAPPα), matching progressive memory decline and cognitive dysfunction [286]. In a large cohort (*n* = 198) of cognitively impaired patients with no dementia, serum brevican levels were significantly lower in those who manifested cerebrovascular burden (i.e., more cerebral infarction/microbleeds) compared to those with lower cerebrovascular burden [31]. Serum brevican detected the presence of cerebrovascular burden with modest sensitivity and specificity (Table 1) [31]. As the epitopes recognized by the antibodies used in this study are not known, it is difficult to compare their findings with the studies detecting ADAMTS‐generated brevican neoepitopes.

By parallel reaction monitoring MS, selected brevican peptides were quantified in the CSF of patients with traumatic brain injury (TBI), finding a significant decrease in peptides located N‐terminally to the ADAMTS cleavage site [287]. This was corroborated by ELISA data in TBI patients showing a correlation between increased CSF brevican levels, which steadily declined after trauma, and unfavorable outcomes [288]. A separate study found that patients with severe TBI had also higher plasma levels of syndecan‐1 compared to controls and in coagulopathic versus non‐coagulopathic TBI patients (Table 1) [50].

## 13 Perspectives and Conclusion

Measuring circulating proteoglycan/proteoglycan fragments provides a readout for the pathogenic processes occurring within the tissue which may not be easily accessible for investigation via biopsy. With few exceptions, such as syndecan‐1, proteoglycans have not been thoroughly investigated as biomarkers of pathological ECM remodeling. As highlighted in the previous sections, the unavailability of selective and sensitive immunological reagents, epitope masking by multiple GAGs attached to the protein core, and limited identification of proteolytic fragments, are some of the reasons behind this untapped potential. When suitable antibodies/ELISAs are available, the amino acid sequence of the epitope used to generate them is not always provided by the supplier, thus further complicating the comparison between different studies which may already vary in cohort ethnicity and size. As soluble proteoglycans may not be present in their full‐length form, epitope definition is crucial. Overall, the lack of suitable, well‐defined antibodies, and their limited characterization is hampering the application of proteoglycans as biomarkers. Proteomics approaches [21] and alternative analytic platforms, for example, those using aptamers [289], will provide competitive alternatives to immunodetection methods only if they improve in robustness and reproducibility while the total costs for their application decrease substantially [290]. Aptamers are short, single‐stranded oligonucleotides with lower manufacturing costs compared to antibodies, but their application in biomarker discovery/validation is currently limited to SomaLogic's SOMAscan platform [291]. The coverage of plasma proteome and sample throughput by SomaLogic is now higher than most MS‐based proteomics [290].

**Figure 3:**
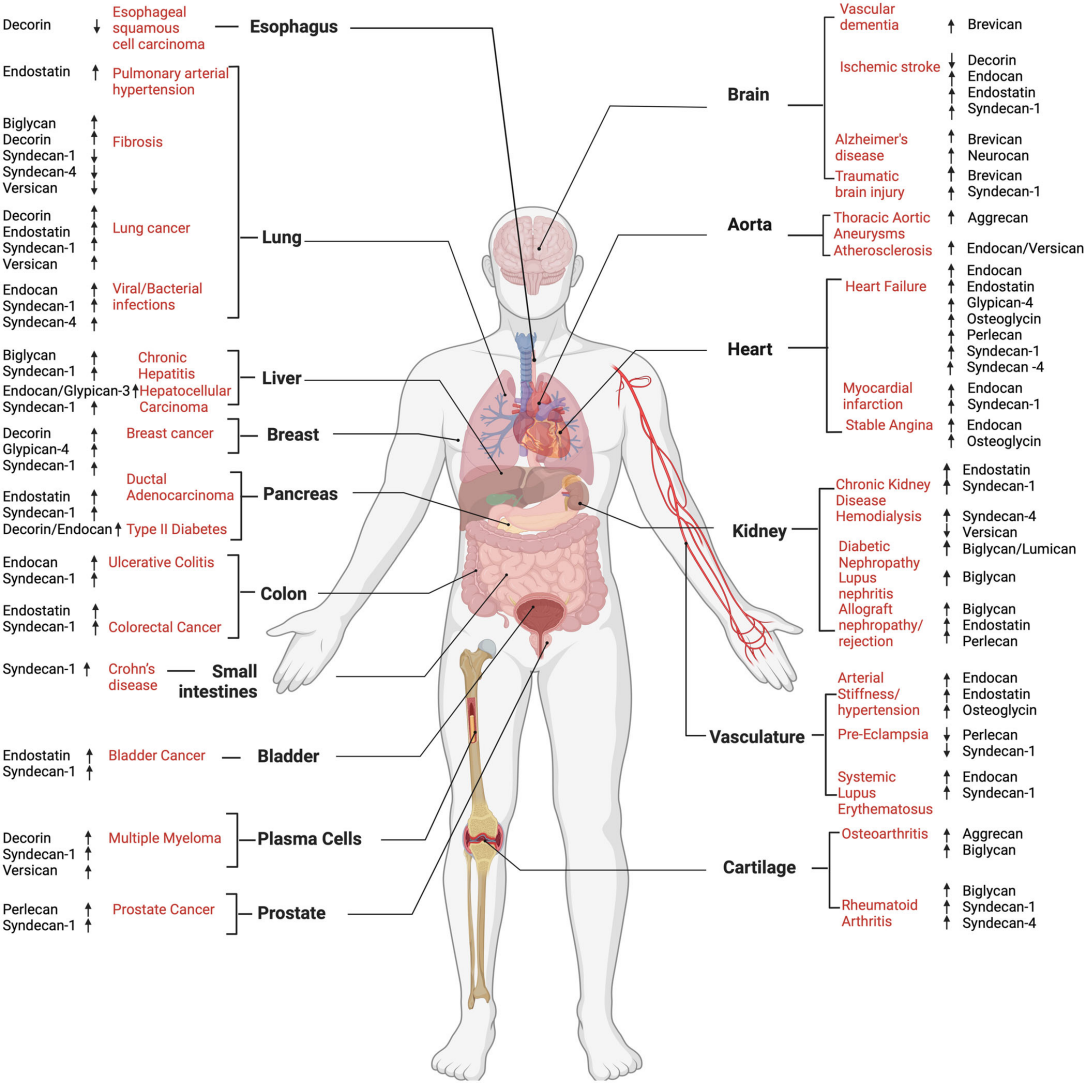
Proteoglycan and proteoglycan fragments as potential biomarkers of pathological ECM remodeling. Some proteoglycans exhibit involvement in various diseases within the same organ, while others are implicated across multiple organs. Note that with the exception of endostatin, all proteoglycan fragments are indicated by the name of the full‐length proteoglycan. Figure created with BioRender.

Another issue with the use of proteoglycans as biomarkers is their widespread and robust expression. Proteoglycans exert a structural, mechanobiological role in most connective tissues [292], therefore variations in their circulating levels may reflect a plethora of pathological processes that may be difficult to pinpoint if they are meant to be used as specific diagnostic biomarkers. This is the case for syndecan‐1 and syndecan‐4, whose increased serum levels are associated with a wide variety of diseases. A valid alternative will be applying proteoglycans as prognostic/theragnostic biomarkers or point‐of‐care testing. For example, there are solid data indicating serum syndecan‐1 as an independent prognostic biomarker in MM [243, 244, 245]. Proteoglycans may also be used to reflect general pathological states in combination with other clinical parameters/markers. Serum syndecan‐1 levels reflect damage of endothelial glycocalyx and may be used to generally predict organ dysfunction and mortality in critically ill patients [293]. Endocan may serve as a marker of systemic inflammation [294], while the angio‐inhibitory fragment of collagen XVIII, endostatin, may be valuable as a prognostic biomarker of cardiovascular and chronic kidney disease [146]. There is an increasing amount of data indicating biglycan as a soluble, noninvasive biomarker of inflammatory renal diseases [15] and liver fibrosis [295]. Brevican and its proteolytic fragments have attracted considerable interest as potential biomarkers for CNS disorders [30, 285]. For other proteoglycans, further studies are needed to draw firm conclusions on their prognostic/diagnostic value (Figure 3).

As proteolytic neopeptides are intrinsically more stable than full‐length proteoglycans [25], analytical assays based on neoepitope antibodies may offer the sensitivity and specificity required by a clinically valuable biomarker. For example, aggrecan ARGSV levels are promising diagnostic and prognostic biomarkers for OA and other joint diseases, with great potential for risk stratification, and response to treatment [70].

VCANM, an MMP‐generated versican fragment, is another successful example of a proteoglycan neoepitope with potential value as a diagnostic/prognostic biomarker for atherosclerosis [83], fibrotic lung disorders [210, 211], and hemodialysis [147]. However, the approach used to develop VCANM, starting from neopeptide identification by LC‐MS/MS and sequence analysis, although powerful, is not intrinsically high‐throughput, and neoepitope antibody generation still represents a considerable bottleneck in the field. Therefore, we do not anticipate that the number of validated neoepitope antibodies against proteoglycans will increase considerably in the next few years. For these reasons, further exploration of the biomarker potential of neopeptides for which specific neoepitope antibodies are already available, such as versikine [196] or Brev‐A [30], is desirable. Given the involvement of versican and ADAMTSs/MMPs in viral infectious diseases [296], determination of both versikine and VCANM levels in infected patients is a gap that needs to be addressed.

Simultaneously measuring circulating levels of neopeptides and the proteases by which they were generated (when this information is available and solidly verified) may be a powerful strategy to increase the positive predictive value of proteoglycans. In some of the studies discussed above, serum syndecan‐1 levels correlated with those of its sheddase MMP7 [253, 264]. Similarly, quantification of serum aggrecan‐ARGSV can be coupled with those of ADAMTS4 or ADAMTS5, and VCANM with MMP8/12, to assess the suitability of these neopeptides as biomarkers of OA and atherosclerosis, respectively. However, the relationship between proteases and their proteolytic products is not simple, as increased soluble levels of proteoglycan fragments may be observed in diseases characterized by proteoglycan accumulation such as IIP, even when protease levels remain stable or decrease [209].

Another important point is that some proteolytic proteoglycan fragments act as matrikines, for example, versikine [193], a 32‐mer aggrecan fragment [64], endorepellin, the angiostatic C‐terminal fragment of perlecan [297], and endostatin, the antiangiogenic C‐terminal fragment of collagen XVIII [298]. As such, their importance is not limited to their ability to act as a readout for pathological processes occurring within the tissue, but they can disclose the mechanism intrinsic to the pathological process itself. Again, very little is known about the biological activity of most soluble proteoglycan fragments as, due to the lack of a systematic approach to characterize their effect on cells, this is frequently left to serendipity.

So far, none of the proteoglycans/proteoglycan fragments discussed in this review have reached the bedside. Divergent results between different studies, low specificity and sensitivity (Table 1), small study size, lack of adequate clinical trials, and replication in independent cohorts, are among the reasons behind the lack of success. However, there is room for optimism. ARGSV‐aggrecan may be implemented into OA clinical practice in the near future. This fragment was initially identified in 1992 [299], thus requiring more than 30 years before testing in clinical trials [72]. The advent of more sophisticated LC‐MS/MS approaches for identifying cleavage sites [192] and the accessibility of repositories such as the PeptideAtlas [300], from which it is possible to match in vitro generated peptides with those detected in human samples, are accelerating the discovery and validation of soluble proteoglycan fragments. The increasing accuracy of LC‐MS in quantification of peptide levels may provide more solid rationale and success in (neoepitope) antibody development for measuring biomarker candidates in the clinic. In the case of lethal diseases characterized by a lack of biomarkers or where existing biomarkers have poor positive predictive value, such as TAA [301], proteoglycans, when used alone or in combination with other general markers, may indeed provide the long sought “Holy Grail” [302].

## Author Contributions

**Marsioleda Kemberi:** conceptualization, writing–original draft, writing–review and editing. **Alexander F. Minns:** conceptualization, writing–original draft, writing–review and editing. **Salvatore Santamaria:** conceptualization, writing–original draft, writing–review and editing, funding acquisition, project administration.

## Conflicts of Interest

The authors declare no conflicts of interest.

## Data Availability

Data sharing is not applicable to this article as no new data were created or analyzed in this study.
